# Examination of eating disorder risk among university marching band artists

**DOI:** 10.1186/s40337-021-00388-7

**Published:** 2021-03-10

**Authors:** Nancy A. Uriegas, Dawn M. Emerson, Allison B. Smith, Melani R. Kelly, Toni M. Torres-McGehee

**Affiliations:** 1 University of South Carolina, Blatt PE Center 212, Columbia, SC 29208 USA; 2 University of Kansas, 1301 Sunnyside Avenue, Robinson 161, Lawrence, KS 66045 USA

**Keywords:** Disordered eating behaviors, Behavioral health, Marching arts, Performing arts medicine

## Abstract

**Background:**

Marching band artists are a physically active population, composed of approximately 27,000 people in the United States. University marching band artists face many of the same physically active demands and mental stressors as student athletes, potentially predisposing them to injury, illness, and risk for eating disorders (EDs). The purpose of this study was to examine ED risk across sex in university marching band artists, and to determine the type of risk based on the Eating Disorder Inventory-3 (EDI-3) and Eating Disorder Inventory-3 Symptom Check List (EDI-3 SC). A secondary aim examined marching band artists and pathogenic weight control behavior use across sex.

**Methods:**

This was a cross-sectional study. A total of 150 marching band artists (female: *n* = 84, male: *n* = 66, age = 19.9 ± 1.1 years) from three National Collegiate Athletic Association Division I university marching bands participated in the study. We screened for ED risk using the EDI-3, and the EDI-3 SC.

**Results:**

Overall, marching band artists were at risk for EDs, using only the EDI-3, 45.3% (*n* = 68) were at risk, with females at significant higher risk than males [χ^2^ = 5.228, *p* = .022]; using only the EDI-3 SC, 54% (*n* = 81) were at risk and no significant differences were found across sex. Overall, 48% of all participants reported dieting and 20.7% engaged in excessive exercise to control weight. Significant differences were found between sex and purging to control weight [χ^2^ = 3.94, *p* = .047] and laxative use [χ^2^ = 4.064, *p* = .044], with females engaging in behavior more than males.

**Conclusions:**

Eating disorder risk was prevalent for both female and male marching band artists, with females displaying higher risk for EDs than males. Furthermore, marching band artists are engaging in pathogenic behaviors to control their weight. Healthcare providers (e.g., physicians, athletic trainers, physical therapist, dietitians, etc.) working in this setting should be aware of the risk factors displayed in marching band artists, and be able to provide education, prevention, and clinical interventions to this population. Additionally, marching band administrators should be aware of all medical risk factors and the benefit of having a healthcare provider (e.g., athletic trainer) to oversee the healthcare and wellness of marching band artists.

## Plain English summary

Marching band artists are a physically active population that faces similar physical and mental stressors as student athletes. The combination of rigorous physical activity, increased stress, and lack of medical oversight predisposes marching band artists to injury, illness, and mental health conditions including eating disorders. This study looks at the prevalence of eating disorder risk in university marching band artists using a multidimensional approach of eating disorder risk and psychosocial components (e.g., low self-esteem, personal alienation, interpersonal insecurity, emotional dysregulation, etc.). The results of this study found that eating disorder risk is highly prevalent in this population and can be attributed to the psychosocial components of eating disorders and pathogenic behaviors including dieting and excessive exercise to control weight.

## Background

The marching arts is a popular activity across the United States; in 2015 it was reported to have approximately 27,000 participants [1]. Marching arts is highly popular throughout the high school years, but many musicians continue marching through college as an extracurricular activity or as part of their major, if pursuing a music degree. Preparation for performance season typically includes introduction to fundamentals of music, marching styles, and placement drills making rehearsals lengthy and strenuous [2]. In general, performing artists (i.e., marching artists, dancers, aerial performers, actors, singers) are involved in highly structured, intense, and achievement-oriented activities. The 2018 Physical Activity Guidelines for Americans recommends 150–300 min of moderate-intensity activity for substantial health benefits [3]. While many may say marching band is not sufficient physical activity, an average of 6.5 h (390 min) per week (excluding performances) is spent doing physical practice with instruments or flags [4]. Furthermore, results from a study examining energy cost in marching band determine marching band met the criteria for moderate activity in adolescents and overall does contribute to physical activity [5]. Additionally, marching artists face mental stressors associated with performance anxiety, as many of them feel very critical and worry about their performance and constantly feel nervous [6, 7]. Therefore, marching artists are at risk for medical and mental health problems. Similar to Fall sport student athletes (football, soccer, and cross country), marching band artists are exposed to high temperatures and humidity and face similar physically active demands and mental stressors [8], that can lead to decreases in physical and mental performance. Additionally, similar to student athletes, artists lack knowledge on proper hydration and nutrition and have to manage stressors associated with class, rehearsal times, sleep deprivation, and long travel hours [8]. Given the increase in physical activity performed by marching band artists [4, 5], the combination of physical and mental stressors and decreased healthcare knowledge place marching band artists at risk for injury, illness and inadequate energy availability (energy deficiency) with or without risk for eating disorder (ED). However, unlike the competitive athletic population, marching bands typically do not have any allied healthcare professionals, such as a team physician or an athletic trainer, overseeing their care to prevent and/or recognize signs and symptoms for injuries, illnesses, and mental health conditions, including EDs.

Eating disorders are serious mental illnesses characterized by the persistent disturbance of eating or eating-related behaviors resulting in altered consumption or absorption of food and psychosocial and physical health impairments [9]. They can be a result of multiple factors including disordered and abnormal eating and compensatory behaviors, including restrictive eating, fasting, frequently skipped meals, diet pills, overeating, binge-eating and then purging (e.g., vomiting, excessive exercise, misuse of laxatives and medications, etc.). Elevated prevalence has been estimated across college students in the United States, with 2.2–30.5% of the studied population screening positive for an ED, with gradual increases over recent years [10–15]. Furthermore, 25.9–46.6% of US college students screened as “high risk” for an ED [11, 12]. While at times performing artists are not considered traditional athletes because of the differences in physical demands, similarities exist, and their demands include an aesthetic component during performances. Thus, marching artists potentially face some of the same risk factors for EDs as athletes. It is well researched and known the athletic population is at a higher risk for EDs compared to non-athletes [16–18]. Female athletes are more predisposed, especially those in aesthetic sports (e.g., gymnastics, cheerleading, swimming, dance) but approximately 10% of all ED cases are seen in males [19–21]. Similar to college students, ED prevalence in the athletic population has been on the rise since the 1990s and increased nearly by 10% in approximately 10 years [17, 18, 22, 23]. Aesthetics or “lean” sports, in which a thin/lean body or low body weight is perceived as the norm or as an advantage to performance, increases ED risk in athletes, most specifically in females [24].

The performing arts is aesthetically driven, from perfect uniforms/outfits to in unison dance moves, marching, and playing. Appearance is a priority, and for many artists this may put them at risk for EDs. In the marching arts we see an array of artists, including musicians in the horn line or drumline, and auxiliaries which are color guard, dancers, and twirlers, each of which may possess different risk factors or comorbidities of EDs. For example, auxiliary teams are extremely aesthetically driven and composed of predominately females who may be wearing revealing attire. We should also note that performing artists often report depression and anxiety, known comorbidities of EDs; 44% of artists reported depression as a health concern and 22% desired help for the problem [6, 7]. Mental health conditions are widely presented in college students including depression, anxiety, eating disorders, alcohol and other substance abuse problems, self-mutilation, and various other self-destructive and reckless behaviors. Multiple explanations exist for the increase in mental health needs for college students; students may not be prepared for the academic demands and adjustment needed to succeed in college; they face increased demands in managing studies, work, and extracurricular activities [25].

While some literature exists identifying ED prevalence in athletes and college students, to date no current research has been conducted examining the prevalence of EDs in university marching band artists. Therefore, the purpose of this study is to examine the prevalence of ED risk across sex in university marching band artists and to determine the type of risk based on the Eating Disorder Inventory-3 (EDI-3) and Eating Disorder Inventory-3 Symptom Check List (EDI-3 SC). A second aim is to examine the pathogenic behaviors (i.e., binge eating, self-induced vomiting; exercise patterns; use of laxatives, diet pills, and diuretics) used to control or lose weight, across marching band artists. We hypothesize there will be an increased prevalence of ED risk in marching band artists (similar to traditional athletes), compared with community norms and female artists will have a higher prevalence compared to males.

## Methods

### Study design

We utilized a cross sectional study design. The study is part of a larger field study and web-based survey examining exertional heat illness risk factors and energy needs in marching band. The portion used for this study was descriptive in nature and used the web-based survey developed from previously validated instruments for quantitative analysis. To best assess both eating attitudes, co-morbid psychological constructs, and eating behaviors, we utilized both the Eating Disorder Inventory − 3 (EDI-3) and the EDI-3 Symptoms Checklist.

### Ethics

Ethics approval for this study was approved by both the University of Kansas (IRB-STUDY00142728) and the University of South Carolina’s (IRB-Pro00081581) Institutional Review Boards. All participants completed an online informed consent with the opportunity to decline participation.

### Participants

Marching band artists were recruited for the study via in-person briefings at a marching band rehearsal by a member of the research team or band director. A total of 182 marching band artists began the study and 150 completed the study, yielding a relative completion rate of 82.4% (female: *n* = 84, male: *n* = 66,) age: (19.9 ± 1.1 years) from 3 NCAA Division I university marching bands. To be included in the study, all participants were enrolled in their respective university’s marching band and between the ages of 18–30 years of age. No exclusion criteria were used for participation in the present study.

### Instrumentation

#### Demographic and baseline medical history questionnaire

A questionnaire was used to acquire basic personal, demographic, and previous medical history data. The information collected included age, education level, sex, ethnicity, years of experience in marching band, and primary instrument/section. Participants also self-reported their height, current weight, lowest weight, highest weight, mental weight, and ideal weight. Mental weight is an individual’s perceived weight if one does not consciously try to control their weight. For example, one may think they will weigh more if they do not exercise or eat healthy to control weight. Eliminating these behaviors such as exercise and eating healthy, may lead someone to feel like they will gain an unrealistic amount of weight.

#### Eating disorder inventory – 3 (EDI-3)

The EDI-3 was administered to screen for ED characteristics and behaviors. This is a 91-item self-reported questionnaire, used by clinicians to identify individuals with disordered eating patterns [26]. A Likert scale is composed of 6 ratings (always, usually, often, sometimes, rarely, never). The EDI-3 consists of 3 ED specific scales and 9 general psychological scales - that are highly relevant to ED patients. It yields 2 main composites, 1 ED specific (Eating Disorder Risk Composite-EDRC) and the overall Psychological Maladjustment composite (GPMC) which consist of 4 general integrative psychological composite constructs (Ineffectiveness-IC, Interpersonal Problems-IPC, Affective Problems-APC, Overcontrol-OC). Examples of statements consisted of: “I think that my stomach is to big”; “I feel ineffective as a person”; and “I trust others”. The EDI-3 is a validated tool and has a high test-re-test reliability of .98 [26]. Two main composites consist of the EDRC (reliability range from .90 to .97 with a median of .95 [26]; current study was .83) and the GPMC (reliability range .93 to .97 and median of .93 [26]; and current study was .824). This instrument is copyrighted by Psychological Assessment Resources, Inc. Permission for use is granted with purchase of inventory. The computer-based scoring program for the EDI-3 was used to assess the outcomes of each individual participants. The software generates individualized score reports with raw scores, T scores, percentiles, and qualitative classifications (Low Clinical, Typical Clinical, Elevated Clinical) for all EDI-3 scales [26]. Clinical ranges are based on percentile ranges for the U.S. Adult Combined Clinical sample (i.e., Low Clinical = 1st to 24th percentile; Typical Clinical = 25th to 66th percentile, Elevated Clinical = 37th to 99th percentile [26]. To account for response style (e.g., irregular, deviant, or anomalous response patterns, etc.; to be considered “at risk” for an ED on the EDI-3, participants had at least two or more composite scores at typical clinical or elevated clinical.

#### Eating disorder inventory – 3 symptom checklist (EDI-3 SC)

The EDI-3 SC is a screening tool used by allied healthcare professionals to identify individuals at risk for EDs and begin the appropriate referral plan. It is a self-reported measure that provides information regarding the frequency of symptoms/pathogenic behaviors (i.e., binge eating, self-induced vomiting; exercise patterns; use of laxatives, diet pills, and diuretics.) The assessment ranges depending on reporting yes or no to behaviors and ranges between 10 and 42 questions. If participant answers yes, they have follow-up questions regarding frequency of behaviors (e.g., not engaged in the behavior in the last 3 months, frequency daily, frequency weekly, frequency monthly). An example would be: have you ever had an episode of eating an amount of food that others would regard as usually large? If the answer is yes, then participants would be asked the following: how old were you when you first had an eating binge and how old were you when you began to binge eat on a regular basis; followed by during the last 3 months how often have you typically had an eating binge. Participants who reported “yes” but did not engage in the behavior in the last 3 months were not considered at risk. The EDI-3 SC is copyrighted by Psychological Assessment Resources, Inc. Permission for use is granted with purchase of inventory. To be considered “at risk” for ED on the EDI-3 SC, participants must meet the criteria for one pathogenic behavior.

### Procedures

After approval from the institutional review boards, participants were notified of the study via in-person briefings at a marching band rehearsal by a member of the research team or band director. Following the briefings, all marching band members were contacted via e-mail. Participants who consented completed the basic demographic and baseline medical history questionnaire, EDI-3, and EDI-3 SC. All data was collected using an online platform, Qualtrics (Provo, UT). The surveys were open for 1 month, a reminder e-mail for participation was sent every 10 days, for a total of 3 reminders.

### Statistical analysis

Data was analyzed using SPSS statistical software (version 26; SPSS Inc., Armonk, NY) with a significance level set at *p* < .05. A priori power analysis was conducted using G*Power statistical software (version 3.1.9.2., Heinrich Heine University, Dusseldorf Germany). Using a moderate effect size at .04, our power calculation indicated a sample size of 124 total participants was needed with estimated power being 0.95. Basic descriptive statistics, including means and standard deviations, were used for demographic information. Overall ED risk was considered if participants had at least two or more composite scores of either “typical clinical” or “elevated clinical” on the EDI-3, and/or met the criteria for at least one pathogenic behavior on the EDI-3 SC. Cross-tabulations and Chi-square analyses were used to examine the proportion of participants classified as “at risk” for an ED, using the EDI-3 only, or the EDI-3 SC only, and across ED Risk Type (EDI-3, EDI-3 SC or both EDI-3 and EDI-3 SC) and across sex.

## Results

A total of 150 university marching band artists from 3 institutions participated in this study. Participants academic status was as follows: 22.7% freshmen/1st year college student (*n* = 34), 31.3% sophomores/2nd year college student (*n* = 47), 22.7% juniors/3rd year college student (*n* = 34), 23.3% seniors/4th year college student (*n* = 35) and ranged from 18 to 23 years of age (mean:19.9 ± 1.1 years). They had participated in marching band 1 year or less (4.7%; *n* = 7), 2–3 years (4%; *n* = 6), 4–5 years (33.3%; *n* = 50), 6–7 years (42%; *n* = 63), or 8 or more years (16%; *n* = 24) and in the following sections: Brass (45.3%; *n* = 68), Woodwinds (36%; *n* = 54) Percussion (7.3%; *n* = 11), or Auxiliaries (11.3%; *n* = 17). Participants identified their ethnicity as White (80.7%; *n* = 121), Hispanic or Latino (2.7%; *n* = 4), Black or African American (5.3%; *n* = 8), American Indian or Alaska Native (0.7%; *n* = 1), or Other (8.7%; *n* = 13). Self-reported physical measures are demonstrated in Table 1.

**Table 1:** Self-reported physical measurements for marching band artists

	Females (***n*** = 84) Mean ± SD	Males (***n*** = 66) Mean ± SD
Age (yrs)	19.9 ± 0.9	19.8 ± 1.3
Current Height (cm)	165.9 ± 7.0	179.3 ± 9.4
Current Weight (kg)	68.7 ± 17.2	81.6 ± 22.6
Highest Weight (kg)	72.6 ± 17.5	87.5 ± 25.0
Lowest Weight (kg)	62.7 ± 14.6	75.7 ± 20.6
Mental Weight (kg)^a^	71.2 ± 18.1	86.4 ± 26.6
Ideal Weight (kg)	61.8 ± 13.8	77.7 ± 15.1
Current Weight-Ideal Weight (kg)	6.7 ± 10.1	3.9 ± 10.7
Current Weight-Mental Weight (kg)	−2.6 ± 5.8	−4.8 ± 7.1
Current BMI (kg/m^2^)	25.0 ± 6.2	25.3 ± 5.9

^a^Mental Weight: Perceived weight if one did not consciously try to control weight

### Eating disorder risk

When assessing combined risk (i.e., EDI-3 Only, EDI-3 SC, or both EDI-3 and EDI-3 SC) 70.7% (*n* = 106) of marching band artists were at risk for EDs with no significant differences across sex (females: 41.3%; *n* = 62 vs. males: 29.3%; *n* = 44; *p* = 0.340). The ED risk per section was as follows: Brass (75%; *n* = 51/68), Woodwinds (63%; *n* = 34/54), Percussion (72.7%; *n* = 8/11), and Auxiliaries (76.5%; *n* = 13/17), no comparisons across section were made due to uneven distribution of sections in our sample. Table 2 demonstrates female and male within sample distribution. Using only the EDI-3, 45.3% (*n* = 68) were at risk, with females having a significant higher prevalence than males [χ^2^ = 5.228, *p* = .022]. Using only the EDI-3 SC, 54.0% (*n* = 81) were at risk and no significant differences were found across sex. For all individuals with ED risk, we categorized their risk type as EDI-3 only, EDI-3 SC only, or being at risk based on both EDI-3 and EDI-3 scores (Table 2); and no significant differences were found across ED risk type and sex.

**Table 2:** Eating disorder risk type in marching band artists. Data is presented “within sex”

	All (***n*** = 150)	Females (***n*** = 84)	Males (***n*** = 66)	***p-***value
	n (%)	n (%)	n (%)	
**EDI-3 Scale Risk**				
EDI-3	68 (45.3)	45 (53.6)	23 (34.8)	0.022
EDI-3 SC	81 (54.0)	47 (56.0)	43 (51.5)	0.588
**Overall, ED Risk (*n*** **= 150)**	106 (70.7)	62 (41.3)	44(29.3)	0.340
**ED Risk Type**				0.108
EDI-3 only	25 (16.7)	15 (17.9)	10 (15.2)	
EDI-3 SC only	38 (25.3)	17 (20.2)	21 (31.8)	
Both EDI-3 and EDI-3 SC	43 (28.7)	30 (35.7)	13 (19.7)	

### EDI-3 primary scales and composite scores

The primary scales and composite scores are displayed in Table 3. It is important to note that the typical clinical category reflect scores and characteristics of patients diagnosed with an ED. Marching band artists in this study had scores ranging from low clinical to elevated clinical across all scales and composites. Elevated clinical scores for ED risk scales were predominately seen in females; while elevated clinical scores for psychological trait scales were seen in both females and males. Overall females endorsed a stronger prevalence for Drive for Thinness (χ^2^ = 8.541, *p* = .014), Bulimia (χ^2^ = 7.215, *p* = .027), Interpersonal Insecurity (χ^2^ = 12.258, *p* = .002), Interpersonal Alienation (χ^2^ = 9.753, *p* = .008), and Maturity Fears (χ^2^ = 7.410, *p* = .025) than males. Furthermore, significant differences were found between sex and the following composites: Eating Disorder Risk (χ^2^ = 7.539, *p* = .023), Interpersonal Problems (χ^2^ = 11.556, *p* = .003], and General Psychological Maladjustment (χ^2^ = 6.962, *p* = .031).

**Table 3:** Eating disorder characteristics among marching band artists. Data is presented “within sex”

	Females (***n*** = 84)			Males (***n*** = 66)			
	Classification			Classification			
	Raw Score^**a**^	Typical Clinical	Elevated Clinical	Raw Score^**a**^	Typical Clinical	Elevated Clinical	***p-***value
	Mean ± SD	n (%)	n (%)	Mean ± SD	n (%)	n (%)	
**Eating Disorders Risk Scale**							
Drive for Thinness	9.0 ± 8.1	17 (20.2)	4 (4.8)	5.2 ± 5.8	5 (7.6)	0 (0)	0.014
Bulimia	5.5 ± 6.6	30 (35.7)	5 (6.0)	3.1 ± 3.5	16 (24.2)	0 (0)	0.027
Body Dissatisfaction	16.4 ± 10.6	20 (23.8)	4 (4.8)	11.1 ± 8.3	10 (15.2)	0 (0)	0.068
Eating Disorder Risk Composite	112.8 ± 27.6	12 (14.3)	4 (4.8)	99.7 ± 18.5	3 (4.5)	0 (0)	0.023
**Psychological Scale**							
Low Self-Esteem	7.0 ± 6.2	22 (26.2)	7 (8.3)	5.6 ± 5.6	11 (16.7)	5 (7.6)	0.348
Personal Alienation	8.1 ± 6.2	25 (29.8)	9 (10.7)	6.6 ± 5.4	16 (24.2)	4 (6.1)	0.380
Interpersonal Insecurity	10.7 ± 6.2	35 (41.7)	24 (28.6)	7.0 ± 5.5)	19 (28.8)	9 (13.6)	0.002
Interpersonal Alienation	7.7 ± 4.9	42 (50.0)	13 (15.5)	6.3 ± 10.6	25 (37.9)	3 (4.5)	0.008
Interceptive Deficits	10.1 ± 8.3	19 (22.6)	12 (14.3)	7.5 ± 6.6)	15 (22.7)	5 (7.6)	0.425
Emotional Dysregulation	4.0 ± 5.0	17 (20.2)	12 (14.3)	2.7 ± 2.9	13 (19.7)	3 (4.5)	0.131
Perfectionism	14.2 ± 5.2	38 (45.2)	29 (34.5)	12.5 ± 5.3	30 (45.5)	18 (27.3)	0.495
Asceticism	6.7 ± 4.8	16 (19.0)	6 (7.1)	6.2 ± 3.8	14 (21.2)	1 (1.5)	0.265
Maturity Fears	10.4 ± 5.9	36 (42.9)	29 (34.5)	7.8 ± 5.1	30 (45.5)	11 (16.7)	0.025
**Composites**							
Ineffectiveness Composite	81.1 ± 19.5	20 (23.8)	10 (11.9)	76.0 ± 17.3	9 (13.6)	5 (7.6)	0.152
Interpersonal Problems Composite	92.4 ± 19.1	41 (48.8)	14 (16.7)	82.6 ± 15.7	20 (30.3)	5 (7.6)	0.003
Affective Problems Composite	84.3 ± 17.6	18 (21.4)	9 (10.7)	78.2 ± 11.6	14 (21.2)	1 (1.5)	0.076
Over control Composite	90.0 ± 14.8	33 (39.3)	11 (13.1)	86.6 ± 12.9	25 (37.9)	4 (6.1)	0.307
General Psychological Maladjustment	404.7 ± 87.3	20 (23.8)	9 (10.7)	369.5 ± 47.2	11 (16.7)	1 (1.5)	0.031

^a^Raw scores calculated from the EDI-3 survey.

### Pathogenic behaviors

The EDI-3 SC examined marching band artists’ pathogenic behavior risk (binging, purging, dieting, etc.) and is displayed in Table 4. Dieting was the most common behavior reported across the entire sample followed by exercise to control weight. Overall, significant differences were found between pathogenic behaviors and sex for purging to control weight (χ^2^ = 3.94, *p* = .047) and laxative use (χ^2^ = 4.064, *p* = .044).

**Table 4:** Eating disorder pathogenic behaviors among marching band artists. Data presented “within sex”

Pathogenic Behavior	All (***n*** = 150)	Females (***n*** = 84)	Males (***n*** = 66)	***p*** -value
	n (%)	n (%)	n (%)	
Dieting	72 (48.0)	43 (51.2)	29 (43.9)	0.378
Exercise	31 (20.7)	18 (21.4)	13 (19.7)	0.795
Binge Eating	28 (18.7)	16 (19.0)	12 (18.2)	0.893
Purging	18 (12.0)	14 (16.7)	4 (6.1)	0.047
Laxatives	5 (3.3)	5 (6.0)	0 (0)	0.044
Diet Pills	7 (4.7)	4 (4.8)	3 (4.5)	0.950
Diuretics	3 (2.0)	2 (2.4)	1 (1.5)	0.707

## Discussion

While different from traditional athletics, the marching arts is a physically demanding activity with similar challenges and health risk as organized sports. The purpose of this study was to examine ED risk among university marching band artists with respect to sex and examine the pathogenic weight control behaviors used by marching band artists. Findings suggest an increased risk for EDs among university marching band artists, with females facing higher risk compared to males. Additionally, and of concern, both female and male marching band artists are engaging in pathogenic behaviors to control their weight, including dieting, exercise, binge eating, and purging.

### Eating disorder risk

Our study used a multidimensional approach (EDI-3, EDI-3 SC, and combination) to assess ED risk across marching band artists, utilizing this approach yielded in 70.7% overall ED risk. However, it is important to note this risk may be inflated with the combination of the 2 instruments. When estimating ED risk across marching band artists using the questionnaires independently from each other (EDI-3: 45.3% vs. EDI-3 SC: 54%) findings reflect lower rates compared to the combined instrument approach. The utilization of independent questionnaires (EDI-3 or EDI-3 SC) still displayed similar and/or higher rates than those in undergraduate students, where “high risk” of EDs was found in 25.9–46.6% of the population [11, 12].

With regards to sex, both females and males were at risk for EDs with females displaying significantly higher prevalence than males. Studies that have examined ED risk in both female and male athletes demonstrate a lower risk, 7–13.5% [16, 17], than marching band artists in the current study. However, we should note that these studies conducted the gold standard of ED diagnosis, which is clinical interviews. Prior to interviews, Sundgot-Borgen et al. [17] and Martinsen et al. [16] conducted screenings using the EDI and EDI-2 respectively. Looking specifically at the results based on the screening, 14.5–25% of the participating athletes were classified at risk for an ED. Our study resulted in 45.3% of marching band artists being at risk utilizing only the EDI-3 tool, yielding close to two times the risk as athletes [16, 17]. We should also note, the evolution of the EDI, as the EDI-3 correctly identified a higher percentage (99%) of patients as positive for ED as compared to the EDI-2 (48%) [27]. Generally speaking, females have higher ED risk than males, but at least 10% of all cases are seen in males [20]. Studies that have looked specifically at the male population demonstrate ED risk in males ranging from 3.2 to 19.2% [16, 17, 19, 28–30], which is consistent with the prevalence of male marching band artists. The findings of this study are consistent with previous literature where female athletes have higher prevalence rates than males [16, 17]. It is important to note there may be some inconsistencies with our results compared to results presented on athletes [16–18, 28–30] One example is the instruments used in our study (EDI-3 and EDI-SC) to assess ED risk. Common instruments used to assess ED risk in athletes are the Eating Attitudes Test (EAT-26) [31], Eating Disorder Diagnostic Scale [32], and the Eating Disorder Examination Questionnaire (EDE-Q) [33]. These instruments range between 22 and 31 questions used to identify eating disorder risk associated with attitudes and behaviors. The EDI-3 is unique because it does assess other psychological comorbidities (e.g., self-esteem, maturity fears, interpersonal alienation, perfectionism, etc.), which may have led to the overall higher risk for EDs compared to athletes.

When looking specifically at female marching band artists, the prevalence rates of at risk are concerning. Typically, female athletes demonstrate increased prevalence of EDs, with rates ranging from 11 to 32.8% [16–18, 22, 28]. ED risk in female marching band artists appears to be higher than these rates, with over half of females being at risk using each tool independently. In the marching arts, a female may participate in the horn line or drum line, but others are in the color guard, dance team, or twirler group which are aesthetically driven and predominately composed of females. Auxiliary unit members have previously been assessed for ED risk, finding 29.7% of them at risk [34]. In our study a high prevalence of ED risk is present in the Auxiliaries section (76.5%; *n* = 13/17) and can be due to the aesthetic nature of these teams within the marching band. However, these findings should be taken with caution because of the very low sample size. Literature suggests athletes in aesthetic or leanness sports have higher ED prevalence rates than those in non-leanness sport [18, 19, 24]. Dancers can be considered aesthetic artists, the rates found in this study for Auxiliaries are closely related to those of dancers, where 69% of them were at risk for EDs [35]. However, we cannot disregard the high percentages of at risk band members in other sections, Brass, Woodwinds, and Percussion. To date, no literature has assessed ED risk in marching band, only the Auxiliary group and it is unknown if they may face some of the same aesthetic concerns and pressures. Special focus should be given to female marching band artists regarding nutrition and EDs, as ED risk may be the beginning of further medical and psychological complications.

### EDI-3 primary scales and composite scores

Our study used a combination of both traditional and comorbid psychological factors related to EDs to assess marching band artists’ ED risk. Evidence suggests that disordered eating attitudes and behaviors are multifactorial with interactions between personal, environmental/cultural, and genetic factors, with various psychosocial components and personality traits as risk factors or comorbidities [36]. While many of our participants did not display traditional ED risk (i.e., Drive for Thinness, Body Image Dissatisfaction, Bulimia), when examining the comorbid psychological risk, they were most prevalent for Interpersonal Insecurity, Interpersonal Alienation, Perfectionism, and Maturity Fears.

The Interpersonal Insecurity scale has a primary focus on assessing the difficulties of conveying one’s personal thoughts and emotions, and the tendency of social inhibition. The Interpersonal Alienation scale assesses disappointment, disassociation, alienation and the distrust in relationships [26]. Previous studies have determined ED patients to have non-assertive interpersonal styles, higher social inhibition, and submissiveness [37, 38]. Furthermore, evidence exists that ED patients also experience social skill difficulties and less social competence than controls and difficulty adjusting in social situations along with social anxiety [39, 40]. Moreover, result indicated that over 50% of our participants had typical or elevated clinical risk for both Interpersonal Insecurity and Interpersonal Alienation. Those who displayed interpersonal insecurity may have thoughts of discomfort, apprehension and are reserved in social situations. In turn, these behaviors may lead to difficulty expressing feelings and personal thoughts with others which may lead to withdrawal and isolation from others. It is also concerning; interpersonal alienation was displayed in half the marching band artists. Interpersonal alienation may lead one to have lack of trust in relationships with people and/or they may feel trapped in relationships. Both of these constructions may lead marching band artists to experience stress and anxiety, which has been previously reported in performing artists which can potentially increase their risk for EDs [6–8].

We observed a large percentage (79.7%) of marching band artists in the typical and elevated clinical classification for Perfectionism. Perfectionism is assessed through evaluating the extent of an individual’s drive to meet high goals and standards, whether that is for personal achievement or to meet others’ expectations [26]. Performing artists often perform in front of large groups and/or at events that are televised or streamed online, this makes artists want to not make mistakes that can constantly be replayed or criticized by their instructors, peers, and friends/family. Perfectionism in the performing arts is widely common, and evidence suggest that it can in fact be a hindering personality trait in the music industry [41]. For marching band artists specifically, the recollection of a perfect performance is extremely meaningful, they are praised for playing the perfect tune while maintaining perfect marching lines and choreography. On the other hand, failed routines are often talked about as examples of what not to do, which may be hindering to the marching band artists’ confidence. At times, the relentless pursuit of perfection can be debilitating psychologically and a contributing factor to mental health conditions including EDs in athletes and performing artists [42, 43]. While many may see perfectionism as an asset in the performing arts, it can also hamper an artists’ health and well-being.

Lastly, we observed a majority of marching band artists displayed typical and elevated clinical classifications for Maturity Fears. Maturity Fear is a desire to return to the security of childhood and the avoidance of developmental demands and weight gains [26]. Given the stressors faced by marching band artists, we can attribute the higher percentages in maturity fears to the avoidance in demands of being college students and performers along with the desire to be a certain weight. While in the later years in college (3rd and 4th year) marching band artists may try to maintain an unrealistic middle school or high school weight which tends to be lower due to maturation. We do observe this with the differences in current and ideal weight, where both females and males self-report their ideal weight as lower than their current weight. Additionally, both females and males have the perception that their weight would be higher if they did not try to control for it, as depicted by their mental weight.

### Pathogenic behaviors

When assessing the pathogenic behaviors to control weight, dieting, excessive exercise, binge eating, and purging were most commonly used by marching band artists. Though females displayed higher percentages than males for all behaviors, only purging was significantly greater. These same pathogenic behaviors are commonly used by collegiate and elite athletes [29, 30, 44]. Specifically looking at females, our study demonstrates higher rates in marching artists than athletes for dieting (51.2% vs 15.6%) and purging (16.7% vs 2.9%), but close similarities in exercise (21.4% vs 25.5%) and binge eating (19% vs 18.6%) [44]. Similar to female athletes, these female artists may experience weight-related pressures, including pressures from instructors, teammates, and oneself to maintain a lean physique for performance, or that of performing in front of hundreds of fans and being on the media. Moreover, revealing attire specific for color guard and dancers may encourage them to engage in behaviors, like dieting and restricting, to rapidly decrease weight. These periods of dieting may also be followed by periods of binge eating or vice-versa.

For male marching band artists, we demonstrate 18.2% engage in binge eating and 6.4% engage in purging, which is consistent with the literature on athletes, 16.7–21.4% for binge eating and 1.6–6.6% for purging [29, 30]. However, rates are two times higher for dieting in male artists as compared to male athletes [29, 30]. Potential reasons for higher rates for dieting may be because athletes in the US may have more resources and access to a dining hall composed of healthier foods planned by a team sport dietitian. Whereas marching band artists who may be trying to adjust their weight may follow fad diets or guidelines found on the internet or from word of mouth and may only have access to university/college dining halls. Male athletes do report higher percentages of exercise to control weight than male artists, but this may be due to the nature of their participation in exercise with the belief that it may increase performance [29, 30].

### Clinical implications

Marching band artists face similar physical and mental stressors as student athletes, with similar predisposition to mental health conditions. While actively participating in marching band rehearsals, performances, and travel, artists also complete the traditional academic workload, ranging from 12 to 20 h per week. Depending on their academic discipline they may need to complete supervised instructional experiences, internships, or full immersion clinical practicums, and some engage in additional physical activity or recreational sports. Ultimately, they must find a balance between being a student and a marching artist and staying physically and mentally healthy. However, while facing similar demands and stressors, an important difference between marching band artists and athletes is that compared to organized athletics most marching band artists do not have medical, psychological, nutritional, or sports performance professionals readily available to discuss best practices of overall health and wellness, including energy intake and exercise expenditure. Having immediate access to healthcare professionals, like a team physician or an athletic trainer, is imperative to this population as these professionals can educate and provide adequate instruction on nutrition and performance to minimize the risk of EDs and injuries and provide immediate referral upon the recognition of mental health conditions. While it is important to take the multidimensional approach, if resources do not permit assessment using multiple forms and/or interviews, clinicians should at least try to use one short ED assessment (e.g., EAT-26, EDE-Q, etc). In this study, using either the EDI-3 or the EDI-SC would also be appropriate, but should be examined independently vs. risk combined as we saw in this study it was over inflated. It is also important to note these assessments only identify risk and if a more thorough evaluation needs to take place, marching band artists should be referred to a physician.

### Limitations and future research

Our study demonstrated increased ED risk in marching band artists; however, some limitations exist. The instruments utilized in this study are self-reported, we assume they were completed entirely, and participants provided accurate and truthful answers. Additionally, these instruments served as an assessment for disordered eating behaviors and ED risk, not as a diagnostic tool. The gold standard for diagnosis would include an interview with the participant; given that participants were anonymous, this interview process was not conducted, and authors were unable to validate our methods of identifying risk for ED compared to the gold standard. Furthermore, participation in our study was limited to 3 U.S university marching bands only. University marching bands vary in performance styles, traditions, and demands and are also associated outside the college setting (e.g., International Drum Corp.). Therefore, these results cannot be generalized to the entire marching band population. Future research should utilize a larger sample size, with a larger distribution of sex and with participants from across states, inclusive to various NCAA divisions, and international marching bands. It is also recommended future research examines college marching band artists to the general college student population to seek any potential risk differences. Researchers should consider conducting follow-up interviews with participants to determine validate the methodology of identifying risk for ED in this study, as well as examine the type of health care available to marching band artists to determine if availability of health care minimizes the risk of EDs and its comorbidities.

## Conclusion

Our study indicates ED risk was highly prevalent for both female and male marching band artists; and females displayed higher risk for ED than males. It is a concern that both female and male marching band artists are engaging in pathogenic behaviors to control their weight, with females displaying higher risk for purging and laxative use. Our findings ultimately reveal crucial information regarding the health and wellness of marching band artists and the importance of medical oversight in this population to be able to educate, prevent and provide adequate treatment or referral for any injury, illness, or mental health condition. Although our findings are novel, yet important, additional research exploring a larger sample size and distribution around the country and world would provide physicians, athletic trainers and other healthcare providers working in this setting more awareness of the risk factors and characteristics of EDs displayed in marching band artists. Additionally, marching band administrators should be aware of all medical risk factors associated with the marching arts and the benefit of having medical oversight, such as a physician and/or an athletic trainer, to oversee the health and wellness of marching band artists.

## Data Availability

The datasets used and/or analyzed during the current study are available from the corresponding author on reasonable requests.

## Abbreviations

ED: Eating disorder
EDI-3: Eating disorder inventory-3
EDI-3 SC: Eating disorder inventory-3 symptoms check
IRB: Institutional review board
NCAA: National Collegiate Athletic Association
